# Knowledge, attitudes, and practices of nurses on dysphagia screening and oral care in acute stroke patients

**DOI:** 10.3389/fneur.2026.1765658

**Published:** 2026-07-21

**Authors:** Mingfang Zhu, Yanan Li, Jing Gong, Lin Ye, Yanhui Wang, Mengke Ma, Yuan Wang, Yue Huang

**Affiliations:** 1 Neurology Department of Henan Provincial People's Hospital, Henan Provincial Key Medicine Laboratory of Nursing, Zhengzhou University People's Hospital, Zhengzhou, Henan, China; 2 Neurology Department of Henan Provincial People’s Hospital, Zhengzhou University People’s Hospital, Zhengzhou, Henan, China

**Keywords:** attitude, dysphagia, knowledge, nurses, oral care, practice, stroke

## Abstract

**Background:**

Acute stroke commonly causes dysphagia, which can delay recovery and worsen nutrition. Nurses detect and treat dysphagia and maintain oral hygiene, but their knowledge, attitudes, and practices (KAP) vary.

**Objective:**

This study aimed to assess nurses’ knowledge, attitudes, and practices regarding dysphagia screening and oral care among stroke patients and to identify factors associated with good attitudes and practices.

**Methods:**

Cross-sectional research comprised 650 tertiary stroke and neurology nurses. Data were collected using a validated self-administered dysphagia screening and oral care knowledge, attitude, and practice questionnaire. Data analysis included descriptive statistics, Pearson’s correlation, and multivariate logistic regression.

**Results:**

Overall, nurses demonstrated good knowledge (mean score = 15.6 ± 3.1), positive attitudes (77.4 ± 9.8), and moderate-to-good practices (73.2 ± 10.5) toward dysphagia management. Though documentation, caregiver education, and formal training were lacking, routine screening and oral care were strongly supported. Knowledge, attitude, and practice showed significant positive associations (*r* = 0.46–0.57, *p* < 0.001). Higher qualifications, career experience, stroke unit experience, dysphagia training, and knowledge were significantly associated with more positive attitudes and practices (*p* < 0.05).

**Conclusion:**

Nurses were knowledgeable and supportive of dysphagia screening and oral care, but inadequate training and institutional support were associated with practical challenges. Improving nursing competency and stroke may benefit from in-service education, standardized screening techniques, and interprofessional teamwork.

## 1 Introduction

Chinese strokes account for roughly one-third of the global stroke burden (1). Age-standardized stroke prevalence is 2,100 per 100,000, with 3.4 million new cases and 2.3 million fatalities in China, Stroke Statistics 2023. China has the highest stroke risk at 39% (2, 3). Age, urbanization, and lifestyle changes increase stroke risk, highlighting public health issues. Dysphagia affects 50–80% of stroke patients immediately and 11–13% after 3 months (4, 5). Swallowing difficulties are associated with an increased risk of hunger, dehydration, hospitalization, death, and impaired function. Acute stroke treatment requires precise and early dysphagia screening (6, 7). All stroke patients should undergo bedside swallowing examinations within 24 h, according the CSA and NHC. Oral hygiene is ignored, and 60–70% of patients require dysphagia screening (8–10). Nurses provide stroke care. They assure swallowing safety, standardize screening procedures like the Standardized Bedside Swallow Assessment (SSA), maintain oral cleanliness, and interact with speech and language therapists to provide rapid treatment (11–13). Studies show associations between nurses’ dysphagia assessment and oral care practices and lower rates of stroke-associated pneumonia (SAP), underlining the importance that nurses have in improving post-stroke outcomes (14, 15). Chinese hospitals differ from national dysphagia screening and oral care nursing recommendations. Despite differences in stroke treatment delivery, dysphagia is a significant and possibly life-threatening stroke consequence globally. Clinical recommendations from the European Stroke Organization and American Heart Association (16), promote dysphagia screening and oral hygiene to avoid aspiration pneumonia and malnutrition. These worldwide guidelines emphasize the necessity of uniform nursing practices in dysphagia treatment throughout healthcare systems. Multiple centers have linked low training, inconsistent screening, insufficient multidisciplinary teamwork, and attitudes that physicians manage dysphagia with inferior nursing practices (17–19). Similarly, staff shortages, high workload, and limited administrative support are associated with lower adherence to evidence-based nursing practices (20, 21). To bridge recommendations and bedside implementation, nurses must understand KAP for dysphagia screening and oral care (22, 23). Nurses with higher KAP levels may notice swallowing difficulties earlier and enhance patient care. Chinese tertiary hospitals may meet the 2019 Stroke Centre Certification Standards by improving KAP (24). Most research is done by medical and rehabilitation specialists, although nurses detect and prevent early. This study examines Chinese acute stroke nurses’ dysphagia screening and oral care knowledge, attitudes, and practices. Opportunities to improve dysphagia management include understanding educational and organizational shortcomings, training modules, and institutional rules. In China’s constantly changing healthcare environment, nurses’ abilities improve stroke nursing, patient safety, and post-stroke outcomes.

## 2 Materials and methods

### 2.1 Ethical considerations

Ethical approval was obtained from the Ethics Committee of Henan Provincial People’s Hospital, Zhengzhou (No. 2023099). Participants were told of the study’s aims, given anonymity, and requested to sign a permission form before data collection. The research utilized anonymized data.

### 2.2 Study design

A cross-sectional study examined nurses’ dysphagia screening and oral care knowledge, attitudes, and practices (KAP) in acute stroke patients. This design was chosen to demonstrate hospital nursing practices and competence. Data was collected from February to May 2025.

### 2.3 Study setting and participants

The research was conducted at Henan Provincial People’s Hospital, a neurological emergency referral hospital that provides acute stroke treatment. To ensure clinical diversity and professional expertise, 800 stroke care registered nurses were recruited using stratified random sampling, out of which 650 nurses were finalized according to inclusion criteria. Volunteer RNs having at least 6 months of neurology, stroke, or critical care expertise who directly cared for acute stroke patients participated in the research. Those on leave, administrative, or not treating strokes were excluded.

### 2.4 Data collection instrument

This structured, self-administered questionnaire was based on the Chinese Stroke Association, American Heart Association/American Stroke Association KAP instruments, and worldwide stroke care standards. Statistics (2) nurses’ dysphagia risk factors, symptoms, consequences, and screening methods knowledge test (3) Attitude measures nurses’ motivation, attitudes, and importance of screening and oral care on a 5-point Likert scale (1 = strongly disagree to 5 = strongly agree); and (4) Practice measures frequency and consistency. Five neuroscience, nursing, and speech-language pathology specialists evaluated content and context. We examined 30 non-study nurses for clarity and dependability. Cronbach’s alpha values of 0.82 for knowledge, 0.86 for attitude, and 0.88 for practice suggest good internal consistency. Online data collecting protected privacy, standards, and access.

### 2.5 Statistical analysis

Data were examined using IBM SPSS 26.0. Demographic characteristics and KAP scores were analyzed using descriptive statistics, such as mean ± SD for continuous variables and frequencies and percentages for categorical variables. Subgroup mean knowledge, attitude, and practice scores were compared using independent *t*-tests. Knowledge, attitude, and practice scores were correlated using Pearson’s correlation coefficient. Multivariate logistic regression analysis reported adjusted odds ratios (AORs) with 95% confidence intervals for positive attitudes and behaviors. All statistical tests were two-tailed, with a *p*-value < 0.05 indicating significance.

## 3 Results and discussion

### 3.1 Socio-demographic characteristics of nurses

Table 1 shows the age, gender, education, job experience, departmental placement, and oral care or dysphagia training of the 650 study nurses. Of the 650 nurses studied, 43.1% were 25–34, and 27.7% were 35–44. 16.9% were under 25, and 12.3% were over 45. Most participants (73.8%) were women, reflecting the Chinese nurse gender ratio. The average professional qualification for nurses was 49.2% Bachelor of Science in Nursing, 40.0% diploma, and 10.8% Master’s. A balanced mix of junior and experienced nurses was shown by 32.3% having one to 5 years of clinical experience, 27.7% having 6 to 10 years, 18.5% having 11 to 15 years, 13.1% having more than 15 years, and 8.5% having less than 1 year. Most participants worked in medical wards (33.8%), intensive care (24.6%), emergency departments (26.2%), and stroke units (15.4%), and saw acute stroke management. Compared to 36.9, 63.1% of nurses had not, indicating a limited level of formal training among the study population.

**Table 1:** Socio-demographic characteristics of nurses.

Variable (*n* = 650)	Category	Frequency (*n*)	Percentage (%)
Age (years)	<25	110	16.9
	25–34	280	43.1
	35–44	180	27.7
	≥45	80	12.3
Gender	Male	170	26.2
	Female	480	73.8
Qualification	Diploma in Nursing	260	40.0
	BSc Nursing	320	49.2
	MSc Nursing	70	10.8
Job experience (years)	<1 year	55	8.5
	1–5 years	210	32.3
	6–10 years	180	27.7
	11–15 years	120	18.5
	>15 years	85	13.1
Department	ICU	160	24.6
	Stroke unit	100	15.4
	Medical ward	220	33.8
	Emergency	170	26.2
Received dysphagia/oral care training	Yes	240	36.9
	No	410	63.1

Data presented as frequency (*n*) and percentage (%).

### 3.2 Knowledge regarding dysphagia screening and oral care

Nursing knowledge of dysphagia screening and oral care among 650 participants is shown in Table 2. Nurses had a strong understanding of dysphagia management and oral care procedures, with a mean knowledge score of 15.6 ± 3.1. 92.3% of nurses correctly defined their function in detecting swallowing issues, 92.3% advised against oral feeding for unconscious patients, and 91.5% recommended feeding in an upright position (≥90°). Nurses also recognized that oral hygiene is associated with lower bacterial load and reduced infection risk (89.2%) and that dysphagia is linked with malnutrition and dehydration (86.2%). Overall, nurses appeared to comprehend the importance of early screening, oral cleanliness, and adequate feeding. Misconceptions exist in clinical knowledge. 58.5% of nurses thought “thickened liquids completely prevent aspiration” was inaccurate, and 63.1% said “nasogastric feeding eliminates aspiration risk” was wrong. Many nurses may not realize that dysphagia may occur in aware or alert stroke patients, as 64.6% correctly stated that “screening is unnecessary in alert patients” is wrong. Aspiration prevention and screening uncertainty may lead to inconsistent clinical decision-making and patient safety.

**Table 2:** Nurses’ knowledge regarding dysphagia screening and oral care.

Knowledge item (*n* = 650)	Correct *n* (%)	Incorrect *n* (%)	Mean ± SD (score 1 = correct, 0 = incorrect)
Dysphagia is a common complication after an acute stroke.	550 (84.6)	100 (15.4)	0.85 ± 0.36
Early screening helps reduce swallowing-related complications.	520 (80.0)	130 (20.0)	0.80 ± 0.40
The water swallow test is a useful bedside screening tool.	495 (76.2)	155 (23.8)	0.76 ± 0.43
Screening should be performed before the first oral intake.	515 (79.2)	135 (20.8)	0.79 ± 0.41
Poor oral hygiene increases the risk of infection.	565 (86.9)	85 (13.1)	0.87 ± 0.34
Thickened liquids completely prevent aspiration *(False)*.	380 (58.5)	270 (41.5)	0.59 ± 0.49
Silent aspiration can occur without coughing.	435 (66.9)	215 (33.1)	0.67 ± 0.47
Nasogastric feeding eliminates aspiration risk *(False)*.	410 (63.1)	240 (36.9)	0.63 ± 0.48
Facial weakness may indicate possible dysphagia.	500 (76.9)	150 (23.1)	0.77 ± 0.42
Nurses play a vital role in the early detection of swallowing difficulties.	600 (92.3)	50 (7.7)	0.92 ± 0.27
Dysphagia can lead to malnutrition and dehydration.	560 (86.2)	90 (13.8)	0.86 ± 0.35
Oral care reduces bacterial load and infection risk.	580 (89.2)	70 (10.8)	0.89 ± 0.31
Screening should be repeated if the patient’s condition changes.	500 (76.9)	150 (23.1)	0.77 ± 0.42
Speech therapists are essential members of the dysphagia management team.	510 (78.5)	140 (21.5)	0.79 ± 0.41
A wet or gurgly voice after swallowing suggests swallowing difficulty.	470 (72.3)	180 (27.7)	0.72 ± 0.45
Tongue control is important for safe swallowing.	455 (70.0)	195 (30.0)	0.70 ± 0.46
Screening is unnecessary in alert patients *(False)*.	420 (64.6)	230 (35.4)	0.65 ± 0.48
Maintaining oral hygiene is essential in dysphagia care.	550 (84.6)	100 (15.4)	0.85 ± 0.36
Unconscious patients should not be given oral feeding.	600 (92.3)	50 (7.7)	0.92 ± 0.27
Feeding should be performed in an upright position (≥90°).	595 (91.5)	55 (8.5)	0.92 ± 0.28
Total knowledge score (mean ± SD)			15.6 ± 3.1

Each correct answer = 1 point; maximum score = 20. Higher scores indicate greater knowledge. “(False)” denotes items intentionally phrased as incorrect; recognizing them as false was considered a correct response.

### 3.3 Nurses’ attitudes toward dysphagia screening and oral care

Table 3 shows nurses’ thoughts on oral care and acute stroke dysphagia screening. Nurses’ views towards dysphagia screening and oral hygiene during stroke therapy were good (grade: 77.4 ± 9.8 out of 100). Employees strongly agreed that dental care is as important as other nursing tasks (88.1%) and that stroke therapy should include dysphagia screening (88.9%). 85.9% said nurses should check for dysphagia to be professional. The mean score of 3.83 ± 1.00 suggests modest self-confidence and skill improvement potential, despite 72.3% of respondents trusting dysphagia screening. Survey results indicate that regular training and teamwork are beneficial for dysphagia therapy (4.55 ± 0.69 and 4.46 ± 0.72, respectively). Most respondents (92%) said treating dysphagia should be a basic nursing skill, and 93.8% thought nurses should attend seminars, reflecting a positive outlook on professional advancement. Time constraints (48.5%, 2.86 ± 1.17), increased treatment effort (40.3%, 2.87 ± 1.15), and aspiration-related concerns (33.4%) were indicated as screening barriers for dysphagia patients.

**Table 3:** Nurses’ attitudes toward dysphagia screening and oral care.

Attitude statement (*n* = 650)	SA (%)	A (%)	N (%)	D (%)	SD (%)	Mean ± SD
Dysphagia screening should be a routine part of stroke care.	52.3	36.6	6.9	3.2	1.0	4.36 ± 0.82
Oral care is as important as other nursing responsibilities.	48.9	39.2	7.1	3.1	1.7	4.30 ± 0.85
Nurses should take an active role in dysphagia screening.	45.1	40.8	8.9	4.0	1.2	4.25 ± 0.87
I feel confident performing dysphagia screening.	30.3	40.0	15.4	11.1	3.2	3.83 ± 1.00
Lack of time limits my ability to perform screening *(Reverse)*.	20.0	28.5	21.5	23.1	6.9	2.86 ± 1.17
Regular training enhances dysphagia management skills.	62.8	32.0	3.5	1.2	0.5	4.55 ± 0.69
Team collaboration improves dysphagia care outcomes.	57.1	34.6	5.7	1.8	0.8	4.46 ± 0.72
Dysphagia screening is a low priority in stroke care *(Reverse)*.	12.9	20.3	18.9	36.0	11.9	2.57 ± 1.17
Effective oral care supports recovery and patient comfort.	55.1	37.2	5.1	1.7	0.9	4.43 ± 0.74
All stroke patients should be assessed for dysphagia.	61.2	31.8	5.4	1.1	0.5	4.52 ± 0.68
Institutional support is essential for maintaining care quality.	49.8	41.5	6.6	1.4	0.7	4.38 ± 0.77
Oral care should be emphasized as part of routine nursing care.	51.7	39.4	6.0	2.0	0.9	4.39 ± 0.79
Fear of aspiration prevents me from feeding patients *(Reverse)*.	14.6	24.8	23.7	28.0	8.9	2.89 ± 1.18
Dysphagia management should be recognized as a core nursing skill.	57.2	34.8	6.0	1.2	0.8	4.46 ± 0.74
I am motivated to enhance my knowledge of dysphagia care.	53.4	38.3	6.0	1.7	0.6	4.42 ± 0.76
Workshops and seminars on dysphagia should be mandatory for nurses.	58.9	34.9	4.0	1.2	1.0	4.49 ± 0.74
Providing dysphagia care increases nursing workload *(Reverse).*	16.5	23.8	19.1	31.4	9.2	2.87 ± 1.15
Early dysphagia screening supports faster stroke recovery.	50.6	38.6	8.0	2.0	0.8	4.36 ± 0.81
Oral care should be provided at least twice daily.	54.9	36.2	6.0	2.0	0.9	4.41 ± 0.78
Proper dysphagia care reflects the overall quality of nursing practice.	59.2	33.1	5.2	1.5	1.0	4.48 ± 0.76
Total attitude score (mean ± SD)						77.4 ± 9.8 (out of 100)

Likert scale: SA, strongly agree; A, agree; N, neutral; D, disagree; SD, strongly disagree. Responses scored 1–5 (strongly disagree = 1 to strongly agree = 5); reverse-coded items adjusted. Higher scores = more positive attitude.

### 3.4 Nurses’ practices regarding dysphagia screening and oral care

In Table 4, nurses provide oral care and assess for dysphagia during acute stroke treatment. A mean score of 73.2 ± 10.5 (out of 100) indicated moderate-to-good practice among participants. Most nurses examine dysphagia and feeding safety periodically. Most (83.7%) examine swallowing before administering oral fluids (4.29 ± 0.90), and 84.8% monitor coughing or choking during feeding (4.33 ± 0.89), reflecting high adherence to safety-oriented feeding practices. While 87.2% feed patients in an upright position (4.43 ± 0.84), 89.0% assure head elevation during and after feeding (4.48 ± 0.80), these practices were associated with safer feeding routines. At least 79.5% of nurses give oral hygiene twice daily (4.19 ± 0.96), and 81.2% provide mouth care before and after feeding (4.20 ± 0.94). Although fewer nurses reported using oral care instruments as brushes or swabs (3.99 ± 1.03), 76.3% regularly check for food retention (4.15 ± 0.96). Moderately good documentation and communication. 70.9% document screening results consistently (3.90 ± 1.08), and 84.6% report swallowing hazards to the following shift (4.29 ± 0.89). Interprofessional teamwork is evident as 78.3% refer suspected dysphagia cases to experts (4.12 ± 0.97). Attendance at dysphagia-related seminars or training sessions (3.52 ± 1.25), where only 56.4% indicated frequent participation, and avoidance of straw feeding (3.86 ± 1.11), were identified as areas for improvement.

**Table 4:** Nurses’ practices regarding dysphagia screening and oral care.

Practice item (*n* = 650)	Always (%)	Often (%)	Sometimes (%)	Rarely (%)	Never (%)	Mean ± SD
Assess swallowing before giving oral fluids.	52.6	31.1	10.3	4.6	1.4	4.29 ± 0.90
Use bedside tests before meals.	41.2	33.8	15.4	6.6	3.1	4.03 ± 1.02
Observe for coughing/choking during feeding.	55.7	29.1	9.4	4.0	1.8	4.33 ± 0.89
Feed only in the upright position.	60.9	26.3	8.6	3.1	1.1	4.43 ± 0.84
Document screening results.	38.0	32.9	15.1	9.1	4.9	3.90 ± 1.08
Refer suspected dysphagia cases to specialists.	44.8	33.5	13.8	5.4	2.5	4.12 ± 0.97
Provide oral care twice daily.	49.5	30.0	13.2	5.1	2.2	4.19 ± 0.96
Use proper oral care equipment (brush, swabs).	40.3	35.5	14.0	6.9	3.3	3.99 ± 1.03
Check for food retention in the mouth.	46.6	32.8	12.6	5.7	2.3	4.15 ± 0.96
Avoid feeding when the patient is drowsy.	56.8	30.5	8.5	3.1	1.1	4.38 ± 0.85
Elevate the head during and after feeding.	63.5	25.5	7.4	2.3	1.3	4.48 ± 0.80
Educate caregivers about safe feeding.	39.1	36.0	14.6	7.1	3.2	3.97 ± 1.06
Suction secretions when necessary.	53.5	30.8	9.4	4.3	2.0	4.29 ± 0.90
Provide mouth care before and after feeding.	47.7	33.5	12.6	4.5	1.7	4.20 ± 0.94
Use thickened fluids when indicated.	41.2	31.5	15.8	7.1	4.5	3.97 ± 1.08
Avoid straw feeding in dysphagic patients.	36.0	32.8	18.0	8.3	4.9	3.86 ± 1.11
Record oral intake daily.	45.7	30.5	14.5	6.6	2.8	4.09 ± 1.00
Clean dentures regularly.	43.1	33.2	15.4	6.0	2.3	4.08 ± 0.99
Attend dysphagia-related training/workshops.	28.9	27.5	20.0	14.5	9.1	3.52 ± 1.25
Communicate swallowing risks to the next shift.	51.4	33.2	10.2	3.7	1.5	4.29 ± 0.89
Total practice score (mean ± SD)						73.2 ± 10.5 (out of 100)

Practice items rated 1–5 (never = 1 to always = 5). Higher scores denote more frequent and appropriate practice (Likert scale: Always/often/sometimes/rarely/never).

### 3.5 Correlation between knowledge, attitudes, and practices regarding dysphagia screening and oral care

Table 5 shows strong positive correlations between nurses’ dysphagia screening and oral care knowledge, attitudes, and practices. A moderate positive correlation (*r* = 0.46, *p* < 0.001) suggests that nurses with more knowledge have more positive attitudes towards dysphagia management. A moderate positive correlation (*r* = 0.52, *p* < 0.001) suggests that better-informed nurses are associated with higher levels of dysphagia screening and oral care practices. A strong correlation (*r* = 0.57, *p* < 0.001) indicates that more positive professional attitudes are associated with more consistent use of evidence-based dysphagia care activities. Overall, these results suggest that higher knowledge and more positive attitudes are linked with better reported clinical practices, highlighting associations rather than causal effects.

**Table 5:** Correlation between knowledge, attitude, and practice scores.

Variables (*n* = 650)	*r*-value	*p*-value	Interpretation
Knowledge vs. Attitude	0.46	<0.001	Moderate positive correlation
Knowledge vs. Practice	0.52	<0.001	Moderate positive correlation
Attitude vs. Practice	0.57	<0.001	Strong positive correlation

Pearson’s correlation used. Significance set at *p* < 0.05.

### 3.6 Multivariate logistic regression analysis for factors associated with good attitude and good practice toward dysphagia screening and oral care

A multivariate logistic regression analysis identified independent variables of nurses’ favorable attitudes and practices toward dysphagia screening and dental care (Table 6). Several demographics, educational, and professional characteristics were strongly associated with better outcomes. Nurses with BSc/MSc in Nursing were almost twice as likely to have a good attitude as diploma holders (AOR = 1.92, 95% CI: 1.28–2.88, *p* = 0.002). People with over 10 years of job experience were twice as likely to have a positive attitude (AOR = 2.14, 95% CI: 1.45–3.17, *p* < 0.001). Stroke unit workers had better attitudes (AOR = 1.67, 95% CI: 1.08–2.59, *p* = 0.021), which was associated with greater exposure to dysphagia care. Nurses with formal dysphagia training had an almost threefold higher likelihood of having a positive attitude (AOR = 2.82, 95% CI: 1.93–4.10, *p* < 0.001). A positive knowledge score was substantially associated with a positive attitude (AOR = 3.05, 95% CI: 2.10–4.44, *p* < 0.001). Similar patterns emerged for good practice. Higher qualified nurses (AOR = 1.74, 95% CI: 1.16–2.61, *p* = 0.007) and those with more job experience (AOR = 2.35, 95% CI: 1.56–3.54, *p* < 0.001) were more likely to follow dysphagia screening and oral care procedures. Stroke unit workers had higher practice scores (AOR = 1.58, 95% CI: 1.04–2.39, *p* = 0.032). Training on dysphagia (AOR = 3.05, 95% CI: 2.10–4.44, *p* < 0.001), knowledge (AOR = 2.68, 95% CI: 1.86–3.86, *p* < 0.001), and attitude (AOR = 3.40, 95% CI: 2.25–5.13, *p* < 0.001) were significantly associated with better reported practice. These findings suggest that cognitive and affective factors directly impact behavioral performance. Gender had a minor but statistically significant effect on attitude (AOR = 1.45, 95% CI: 1.02–2.06, *p* = 0.038), suggesting that female nurses had more positive views than male nurses, with no significant association with practice. Age made little difference in attitude or practice.

**Table 6:** Multivariate logistic regression analysis for factors associated with good attitude and good practice toward dysphagia screening and oral care among nurses (*n* = 650).

Variable	Category	Good attitude adjusted OR (95% CI)	*p*-value	Good practice adjusted OR (95% CI)	*p*-value
Age group	≥35 years vs. < 35 years	1.38 (0.94–2.03)	0.10	1.24 (0.86–1.78)	0.25
Gender	Female vs. Male	1.45 (1.02–2.06)	0.038	1.32 (0.95–1.85)	0.10
Qualification	BSc/MSc Nursing vs. Diploma	1.92 (1.28–2.88)	0.002	1.74 (1.16–2.61)	0.007
Job experience	>10 years vs. ≤ 10 years	2.14 (1.45–3.17)	<0.001	2.35 (1.56–3.54)	<0.001
Department	Stroke unit vs. Others	1.67 (1.08–2.59)	0.021	1.58 (1.04–2.39)	0.032
Training on dysphagia	Yes vs. No	2.82 (1.93–4.10)	<0.001	3.05 (2.10–4.44)	<0.001
Knowledge score	Good vs. Poor	3.05 (2.10–4.44)	<0.001	2.68 (1.86–3.86)	<0.001
Attitude score	—	—	—	3.40 (2.25–5.13)	<0.001

OR, odds ratio; CI, confidence interval. Both models adjusted for age, gender, qualification, job experience, department, and training. Logistic regression.

## 4 Discussion

Dysphagia in acute stroke is associated with nurses’ knowledge, attitudes, and behaviors toward screening and oral care. Less than 40% of Chinese nurses are trained in oral hygiene and dysphagia (25, 26). South Korean and Japanese nurses received insufficient post-stroke dysphagia training, according to international research (27). According to this research, younger, early-career nurses may lack specialized training. These data show that Chinese acute stroke nurses require comprehensive training and continuing professional development to improve oral care and dysphagia screening. This study supports previous studies under comparable settings (28). A multidisciplinary team of neurologists, speech-language pathologists, physiotherapists, nutritionists, and occasionally pharmacists treat dysphagia and oral care. Nurses are vital, but their roles and scope of practice differ by country, the results should be evaluated in the context of the Chinese healthcare system while still providing insights into worldwide stroke treatment. The Chinese acute stroke team manages dysphagia and oral hygiene, with nurses providing bedside screening, oral care, patient placement, and monitoring. Assessment, eating planning, rehabilitation, and medication management are supported by neurologists, speech-language therapists, physiotherapists, dietitians, and occasionally pharmacists Experts do FEES and X-rays. China’s nurses are more direct than international norms due to staffing, training, and institutional regulations. A previous research indicated that nurses had moderate to strong dysphagia knowledge but failed to diagnose silent aspiration and understand nasogastric feeding restrictions (29). The mean knowledge score of 15.6 ± 3.1 in this study is somewhat higher than 14.8 ± 3.6 in an earlier Saudi nurse study (28), indicating improved awareness in the present cohort. 80–85% of nurses understood the need for early screening before oral health treatment (30), closely matching the 79.2% in the current trial for the same item. Previous research has shown that nurses overestimate the protective impact of thickened fluids and nasogastric feeding (31, 32). Speech-language pathologist referral delays and management issues may be linked to misconceptions, suggesting the need for ongoing training. Attendance at seminars, in-service training, and multidisciplinary speech therapy was associated with higher adherence to evidence-based dysphagia treatment practices, with a mean score of 75.6 ± 10.2 (25). Only 87% of stroke unit nurses endorsed standard dysphagia screening, compared to 88.9% in the previous research (33). A research revealed that continuous education and interprofessional cooperation enhance nurse screening accuracy and confidence (34). However, time and effort were typically cited as regular screening constraints (35). Dental and dysphagia screening are popular with nurses, but they require institutional support and open protocols to adopt favorable attitudes in clinical practice (22). Nurses like treating dysphagia, but they require continual training, administrative support, and adequate personnel to overcome practical challenges and enhance clinical implementation. Researchers found that while nurses’ dysphagia procedures are acceptable, documentation, advanced management, and training participation need improvement. In this study, a total practice score of 72.5 ± 11.2 suggested reduced recording and referral rates but good feeding-position adherence (28). In addition to upright feeding, almost 80% of nurses noticed aspiration indications (36). Although less than 60% of respondents reported frequent dysphagia testing (mean = 3.90 ± 1.08), this confirms the present results. Despite nurses’ safety knowledge, poor training and workshops impair clinical competence (37). Low workshop attendance (3.52 ± 1.25) highlights the necessity for continued institutional training. Even though nurses are dysphagia specialists, paperwork, caregiver education, and regular oral care need organization and administration (38). The research shows that clinical audits and policy initiatives are needed for high-quality dysphagia therapy. The latest results confirm previous research associating dysphagia to stroke care knowledge, attitude, and practice. The moderately to substantially favorable connection between practice and knowledge (*r* = 0.48–0.55) (39), supporting this conclusion (*r* = 0.52). Nurses’ cognitive understanding was associated with clinical performance, with higher awareness linked to safer feeding and oral care practices (40). Positive attitudes, such as perceiving dysphagia screening as a nursing duty, were associated with higher adherence to interventions (41). Screening and oral hygiene compliance required nurses’ confidence and commitment (42). This research suggests that comprehensive dysphagia education programs that increase attitudes and understanding improve nursing practice. Comprehensive institutional training, clinical mentoring, and multidisciplinary collaboration were associated with higher knowledge, attitudes, and behaviors that may lead to improved, evidence-based stroke treatment for dysphagic patients. Professional training, education, and experience were associated with differences in nurses’ dysphagia management practices, according to studies (43). Diploma holders were less competent and less likely to follow screening standards than postgraduate nurses (44). Participation in organized training programs was associated with greater confidence, more frequent early screening, and oral care adherence (AOR = 3.0) (45, 46). Dysphagia assessments and specialist nurses were common in multidisciplinary stroke care units (47). KAP says knowledge impacts attitude and conduct. Research shows that educational therapies increase dysphagia awareness, motivation, and practice (38, 48). These findings suggest that stroke nurses need dysphagia training, professional development, and institutional support to improve their attitudes and behaviors.

## 5 Limitations

This study, conducted within the Chinese healthcare system, may have limited generalizability internationally. The single-arm cross-sectional design prohibits causal inference, and self-reported data may bias responses. More long-term or objective research is required to confirm these conclusions.

## 6 Conclusion

This research indicated that nurses had moderate to excellent dysphagia screening and oral care knowledge, attitude, and acute stroke treatment practice. Additionally, these domains correlated positively. Study was cross-sectional. Higher credentials, longer employment, stroke unit work, dysphagia training, and knowledge improved attitudes and practice. These results stress nurse competence, focused education, and institutional support. For more consistent dysphagia treatment, hospitals should standardize screening instruments, develop organized and continuing training programs, and promote multidisciplinary teamwork. Administrative and policy reinforcement, research, and quality improvement promote optimal practices and enhance stroke patient outcomes.

## Data Availability

The original contributions presented in the study are included in the article/supplementary material, further inquiries can be directed to the corresponding author.
